# The Helminth Parasite *Heligmosomoides polygyrus* Attenuates EAE in an IL-4Rα-Dependent Manner

**DOI:** 10.3389/fimmu.2020.01830

**Published:** 2020-09-29

**Authors:** Madeleine P. J. White, Chris J. C. Johnston, John R. Grainger, Joanne E. Konkel, Richard A. O'Connor, Stephen M. Anderton, Rick M. Maizels

**Affiliations:** ^1^Wellcome Centre for Molecular Parasitology, Institute of Infection, Immunity and Inflammation, University of Glasgow, Glasgow, United Kingdom; ^2^Clinical Surgery, Royal Infirmary of Edinburgh and University of Edinburgh, Edinburgh, United Kingdom; ^3^Faculty of Biology, Medicine and Health, Lydia Becker Institute of Immunology and Inflammation, University of Manchester, Manchester, United Kingdom; ^4^MRC Centre for Inflammation Research, Centre for Multiple Sclerosis Research, Centre for Immunity, Infection and Evolution, University of Edinburgh, Edinburgh, United Kingdom

**Keywords:** autoimmunity, intestinal nematode, multiple sclerosis (MS), Th2 (type-2) immune responses, cytokine

## Abstract

Helminth parasites are effective in biasing Th2 immunity and inducing regulatory pathways that minimize excessive inflammation within their hosts, thus allowing chronic infection to occur whilst also suppressing bystander atopic or autoimmune diseases. Multiple sclerosis (MS) is a severe autoimmune disease characterized by inflammatory lesions within the central nervous system; there are very limited therapeutic options for the progressive forms of the disease and none are curative. Here, we used the experimental autoimmune encephalomyelitis (EAE) model to examine if the intestinal helminth *Heligmosomoides polygyrus* and its excretory/secretory products (HES) are able to suppress inflammatory disease. Mice infected with *H. polygyrus* at the time of immunization with the peptide used to induce EAE (myelin-oligodendrocyte glycoprotein, pMOG), showed a delay in the onset and peak severity of EAE disease, however, treatment with HES only showed a marginal delay in disease onset. Mice that received *H. polygyrus* 4 weeks prior to EAE induction were also not significantly protected. *H. polygyrus* secretes a known TGF-β mimic (*Hp-*TGM) and simultaneous *H. polygyrus* infection with pMOG immunization led to a significant expansion of Tregs; however, administering the recombinant *Hp-*TGM to EAE mice failed to replicate the EAE protection seen during infection, indicating that this may not be central to the disease protecting mechanism. Mice infected with *H. polygyrus* also showed a systemic Th2 biasing, and restimulating splenocytes with pMOG showed release of pMOG-specific IL-4 as well as suppression of inflammatory IL-17A. Notably, a Th2-skewed response was found only in mice infected with *H. polygyrus* at the time of EAE induction and not those with a chronic infection. Furthermore, *H. polygyrus* failed to protect against disease in IL-4Rα^−/−^ mice. Together these results indicate that the EAE disease protective mechanism of *H. polygyrus* is likely to be predominantly Th2 deviation, and further highlights Th2-biasing as a future therapeutic strategy for MS.

## Introduction

Over one-fourth of the global population are infected with helminth parasites, with the majority of these infections located within resource-poor tropical countries (1); however before sanitation improvements and widespread industrialization occurred within the last century, the prevalence of helminths was likely to be high across the globe. Helminth parasites are widely known to induce a state of host immune hypo-responsiveness that has been associated with a decreased incidence of inflammatory diseases, a concept that is consistent with the original “hygiene hypothesis,” which suggested that increased prevalence of allergy and asthma in industrialized countries is at least in part due to the reduction in parasitic burden (2, 3).

Multiple sclerosis (MS) is a severe degenerative autoimmune disease characterized by lesions in the brain resulting in a range of symptoms including paralysis, loss of vision and co-ordination (4). MS is believed to be driven by autoreactive T helper cells, particularly the Th1 and Th17 subsets, which enter and are re-activated in the central nervous system (CNS) resulting in the recruitment of additional T cells and macrophages to establish inflammatory lesions. These lesions result in loss of myelin, oligodendrocyte destruction and axonal damage and can account for the broad range of symptoms seen in patients with MS (5). While CD4^+^ T helper cells are well-established as important for initiating MS disease; B cells, CD8^+^ T cells, and natural killer cells have also been implicated as drivers of disease pathogenesis (6).

The incidence of MS in developed countries is increasing and a number of reasons have been suggested for this, including a loss of co-evolved helminth infections (7, 8). Striking evidence for helminth-induced protection in MS came from an Argentinian patient cohort that unintentionally acquired gastrointestinal helminth infection with a variety of species. Those infected showed a significantly lower frequency of disease exacerbations and fewer visible lesions on sequential interval MRI scans in comparison to an uninfected MS patient cohort (matched for age, sex, and time since diagnosis of MS) over the same period of time (9). In addition, after 63 months of follow up, a subset of patients within the helminth-infected cohort were given anti-parasitic treatment which lead to an increase in clinical and radiological disease indicating helminth induced regulatory pathways are playing a role in disease reduction (10). Following on from these observational studies several clinical trials were initiated using *Trichiruis suis* and *Necator americanus*, which were chosen due to their favorable infection safety profile. However, while these studies confirmed safety, the efficacy of these trials were mixed, indicating that the context and dose required may differ from patient to patient, and helminth to helminth (11, 12). Ultimately, understanding how helminths modulate the immune system during infection in MS patients will be required if helminth therapy is to be used more effectively in the future.

To further understand the mechanisms by which helminths are able to suppress inflammatory diseases, we look to animal models of infection and disease. Experimental autoimmune encephalomyelitis (EAE) is a mouse model of MS that is induced by priming with myelin peptides and/or protein resulting in CNS-specific pro-inflammatory Th1 and Th17 cells that drive neurodegeneration (13, 14). In this model, disease is dependent on Th1 cytokines such as IL-12, and more so on the Th17-driving cytokine IL-23 (15), as well as GM-CSF and IL-1 (13). In contrast, Th2 cytokines and regulatory cells (Tregs) are known to reduce disease severity (16). Helminths are known to strongly induce Th2 responses and some are associated with increased Treg numbers, therefore it is unsurprising that mice subjected to EAE that are simultaneously infected with helminths show a reduced EAE disease severity compared to non-infected mice, as comprehensively reviewed by other authors (12, 17).

Helminths employ a range of immune suppressive mechanisms to enhance their survival within the host such as expansion of Tregs and myeloid derived suppressor cells or by releasing factors that can suppress anti-helminthic Th2 immunity. These pathways are also capable of suppressing bystander inflammation; for example infection of EAE mice with *Schistosoma mansoni* induced regulatory macrophages capable of modulating CNS inflammation (18), whereas immunizing with egg antigens from either *S. mansoni* or *S. japonicum* suppressed EAE progression by inducing Th2-deviation and IL-4 production, resulting in reduced MOG-specific Th1 and Th17 cytokines (19, 20). In the case of *Fasciola hepatica*, a reduction of EAE disease was attributed to TGF-β-induced suppression of Th1 and Th17 responses and an expansion of Tregs (21). Infection with a native mouse intestinal helminth parasite, *Heligmosomoides polygyrus*, is also associated with an increase in the number of regulatory T cells as well as strong Th2 responses. In part *H. polygyrus* drives Tregs by secreting a protein named *Hp-*TGM that mimics the activity of mammalian TGF-β and is known to induce Tregs *in vitro* (22). This protein is just one of many identified from *H. polygyrus* excretory/secretory products (HES), from which a number of exciting immune modulating proteins have been found.

Previous work has identified that infection with *H. polygyrus* suppresses EAE disease severity when infection begins after onset, however the mechanisms by which this suppression is mediated are not fully defined (23–25). Therefore, this study aimed to identify the role *H. polygyrus* and its excretory/secretory products play in EAE disease suppression, and whether this protective effect is mediated through Th2 immune-deviation or induction of Tregs. We determined that *H. polygyrus* is able to ameliorate EAE disease severity in an IL-4Rα-dependent manner and that protection requires live helminth infection as HES itself is unable to induce a significant amount of disease protection. Furthermore, disease protection is associated with increased Tregs, GATA3^+^ and ST2^+^ cells, reduced RORγt^+^ and IL-17A cell responses, and a lower level of myeloid cell infiltration into the CNS.

## Methods

### Animals

Female inbred C57BL/6, IL-4Rα^−/−^ (26) and Foxp3-GFP C57BL/6 reporter (27) mice were used for experiments aged between 6-14 weeks old. All mice were either bred in-house or sourced from the University of Edinburgh and housed in the University of Glasgow animal facility. All experiments were performed under UK Home Office licence and approved by the University of Glasgow and/or University of Edinburgh Ethical Review boards.

### EAE Immunization

Mice were induced for EAE using previously published protocols (28). In short, an emulsion of MOG_35−55_ (Genscript, USA) was prepared in Complete Freund's Adjuvant (Sigma, USA) and passed through a 19G needle (BD Biosciences, USA) with glass syringe until homogenous and opaque. Mice received 100 μl subcutaneously in each hind limb, followed by 200 ng of pertussis toxin (Sigma) intra-peritoneally (i.p) in 200 μl of pertussis toxin buffer (Triton-X 0.017%, Tris pH 7.4 15 mM, Sodium chloride 0.5 M). On day 2, mice received a repeat dose 200 ng of pertussis toxin i.p. Mice were monitored closely and weighed daily from disease onset, EAE mice were scored as previously published (28), 0 = unaffected; 0.5 = loss of tonicity in the distal region of the tail; 1 = half-tail paralysis; 2 = full tail paralysis; 3 = one hind limb paralysis or severe weakness in both hind limbs; 4 = full hind limb paralysis; and 5 = moribund. If any group in one experiment reached the humane endpoint of severity, all groups were terminated for analysis, between days 19 and 25 post-EAE induction.

### Treatment Regimens

The parasite *Heligmosomoides polygyrus* life cycle was maintained through serial passage of CBA x C57BL/6 F1 mice as previously described (29), and experimental EAE mice received 200 L3 larvae via oral gavage on either the same day as EAE induction or 4 weeks beforehand, as indicated by the Figure legends. *Hp*-TGM was prepared as previously described (22) and 1 μg/mouse was injected intraperitoneally either on days −1, 1, 3, 5 or days 10, 12, 14 as indicated in the Figure legend. Continuous infusion of HES or PBS via ALZET osmotic minipump (Charles River, UK) using 100 μl capacity (model 1004, 28 days). The minipumps were primed by incubation with HES or PBS overnight at 37°C 2 days before surgical insertion as previously described, and shown to be effective at immune system modulation over a 14-day period of release (22).

### Parasite Counts

To assess the parasite burden, intestinal adult worms were counted macroscopically and feces were collected from the mice from day 14 post-parasite infection. The feces were weighed and left to soak in 1 ml of water for at least 1 h at 37°C, or overnight at 4°C, until soft and then mixed with 1 ml of saturated salt solution (0.27 g NaCl per ml of water). After agitation, samples were transferred to McMaster chambers and eggs were counted using a dissecting microscope (Leica, Germany). Fecal counts are expressed as the number of eggs per mg of feces.

### Tissue Isolation and Single Cell Suspension Preparation

After mice were terminated by CO_2_ asphyxiation, spleen and inguinal lymph nodes (iLN) were removed and processed into a single cell suspension by passing the tissues through a 70 μm cell strainer (Greiner Bio-One, Austria), then resuspended in complete culture medium containing Dulbecco's minimal essential medium, supplemented with 100 U/ml penicillin and 100 μg/ml streptomycin, 2 mM L-glutatmine, 10 mM Hepes and 10% heat-inactivated fetal calf serum (FCS) (all from Gibco, USA). Where needed, red blood cells (RBC) were lysed from 2 min at room temperature with 2 ml of RBC lysis buffer (Sigma, USA). Cell viability was calculated using Trypan Blue exclusion (Sigma) and then resuspended at a concentration of 5 × 10^6^ or 1 × 10^7^ for downstream flow cytometry or MOG-restimulation, respectively.

Spinal cords were also harvested following perfusion of the mouse with 20 ml of PBS (Gibco). The tissue was then finely chopped using a scalpel and resuspended in 2.4 mg/ml collagenase type II (Gibco) and left for 30 min in a shaking 37°C incubator. After incubation, breaking up the clumps by repeat pipetting with a P1000 pipet and passing through a 70 μm cell strainer (Greiner Bio-One) then washing the cells and resuspending in a 33% Percoll solution [33% Percoll (GE healthcare, USA), dPBS (Gibco) and HCl to neutralize] and centrifugation at 760 g for 30 min with no brake. The top layer containing myelin was discarded and the cell pellet resuspended in a low volume of PBS before flow cytometric analysis.

### Flow Cytometry Analysis

Single cell suspensions of spleens, inguinal lymph nodes and spinal cords were stained using live/dead dye and stained with fluorescent antibodies before being run on a Celesta or LSRII flow cytometer (BD Biosciences, USA). Fcγ receptors were blocked by the addition of purified anti-CD16/CD32 (eBioscience, USA) as well as the blocking of non-specific antibody binding by the addition of polyclonal rat IgG (Sigma, USA), before staining with antibodies to stain extracellular surface markers including: rat anti-CD4-PerCP/Cy5.5 (clone GK1.5), rat anti-CD45- PE/cy7 (clone 30-F11), rat anti-CD3-BV711 (clone 17A2), rat anti-PD-1-PE (clone 29F.1A12, Biolegend, USA) and rat anti-ST2-AF488 (clone RMST2-2) (eBioscience). After extracellular staining was completed, the cells were fixed and permeabilised using the Foxp3 transcription factor buffer kit (eBioscience) as per manufacturer's guidelines. Antibodies used to stain transcription factors included: rat anti-RORγt-PE (clone AFKJS-9), rat anti-Foxp3-eF450 (clone FJK-16s, eBioscience) and rat anti-GATA3-AF488 (clone 16E10A23, Biolegend). After acquisition on the flow cytometer, data was analyzed using FlowJo (BD Biosciences, USA).

### ELISA

Splenocytes were resuspended in complete tissue culture media at a concentration of 1 × 10^6^ cells per well in a 96 well plate (Corning, USA) in the presence of MOG_35−55_ peptide (Genscript, USA) at concentrations ranging between 0.3 and 30 μg/ml for 72 h at 37°C in 5% CO_2_. The plates were centrifuged at 400 g for 5 min to pellet the cells and supernatants removed and immediately stored at−80°C until ELISA analysis. Interleukin (IL-) 10, IL-4, IL-5, IL-17A and interferon-γ (IFN-γ) sandwich ELISAs were performed as per manufacturer's instructions (eBioscience, USA).

### Statistical Analysis

All graphs and data analysis were performed using Prism (GraphPad, USA).

## Results

### Infection With *H. polygyrus* at Day 0 Reduces EAE Disease Severity

**Figure 1:**
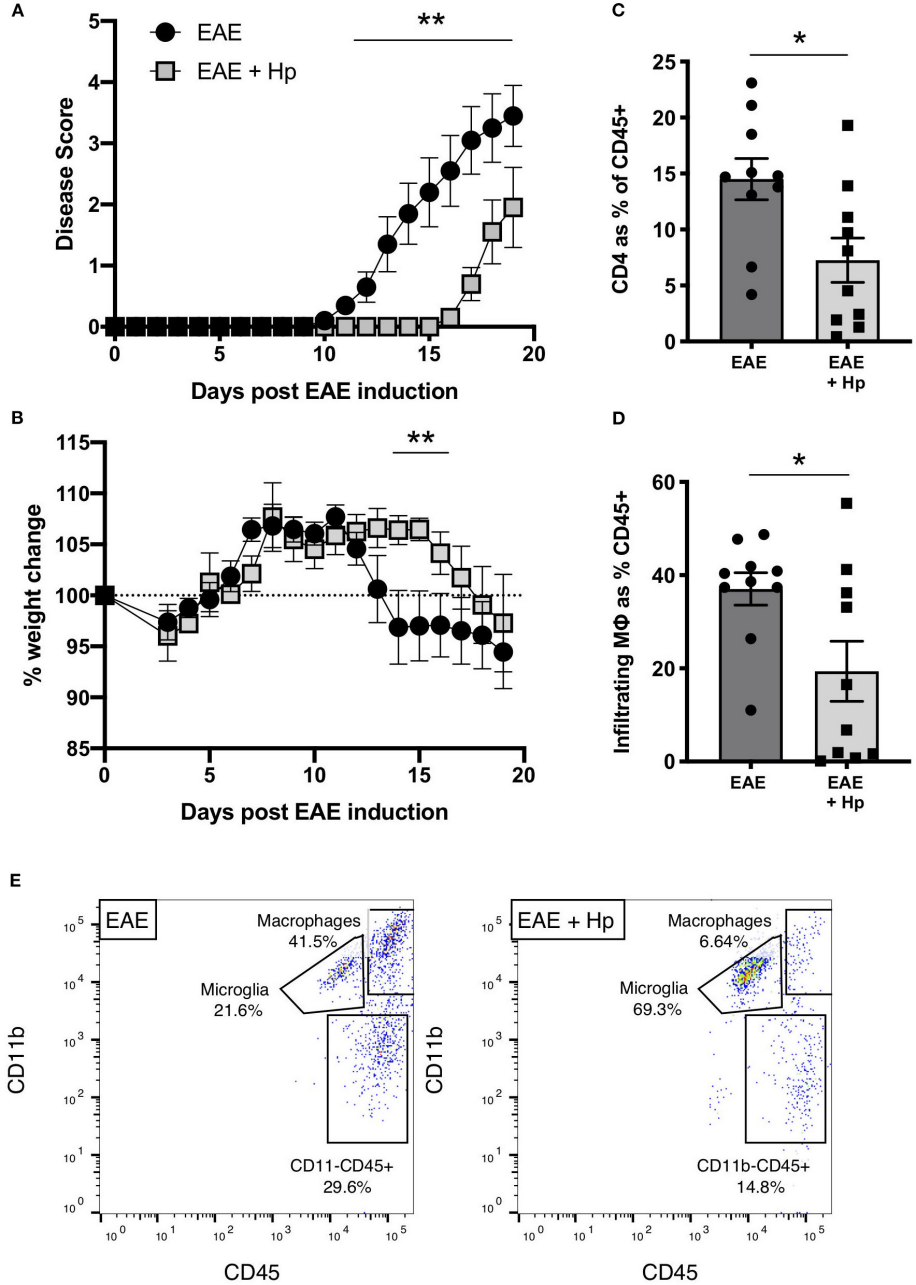
*H. polygyrus* suppresses EAE disease in mice infected on the day of immunization. Female C57BL/6 mice were immunized for EAE on day 0 and were either left untreated (EAE), or received 200 L3 *H. polygyrus* larvae also on day 0 (EAE + Hp). **(A)** Disease score (see Methods). **(B)** Weight change. **(C)** Spinal cord CD4^+^ T cell infiltration at euthanisation on day 19. **(D)** Spinal cord macrophage infiltration (CD11b^+^CD45^hi^ cells as gated in **(E)** and detailed in Supplementary Figure 1) at euthanisation on day 19. **(E)** Within the gate of live single leukocytes, CD45^+^ spinal cord cells were further gated into non-myeloid cells (CD45^hi^CD11b^−^), microglia (CD45^int^CD11b^+^), and infiltrating macrophages (CD45^+^CD11b^+^) as detailed in Supplementary Figure 2. Plots shown are taken from individual mice with median values in **(D)**. Data are pooled from two independent experiments, with a total *n* = 10, and show arithmetic means and standard errors. Data were analyzed by 2-way ANOVA with Sidak's multiple comparisons test **(A,B)** or by Mann–Whitney non-parametric test **(C,D)**, *p < 0.05, **p < 0.01.

Previous works have shown that infection with 200 L3 *H. polygyrus* larvae during the chronic phase of EAE is able to suppress clinical disease within 3–6 days post infection, indicating that the L4 larvae stage is able to attenuate disease symptoms and pathology (23, 24). To investigate further the impact of adult *H. polygyrus* on EAE, mice were infected on the same day as the EAE immunization protocol, which results in adult worms being present in the small intestine during the onset of EAE disease (days 10–14). Mice infected with *H. polygyrus* showed a significant amelioration in disease severity, and a 4-day delay in weight loss (Figures 1A,B). Additionally, the reduction in disease symptoms in *H. polygyrus-*infected mice correlated with reduced CD4^+^ T cell (Figure 1C, Supplementary Figure 1A) and macrophage (Figures 1D,E, Supplementary Figures 1B **and** 2) infiltration into the spinal tissue, confirming that parasite infection impedes EAE disease progression in the CNS.

**Figure 2:**
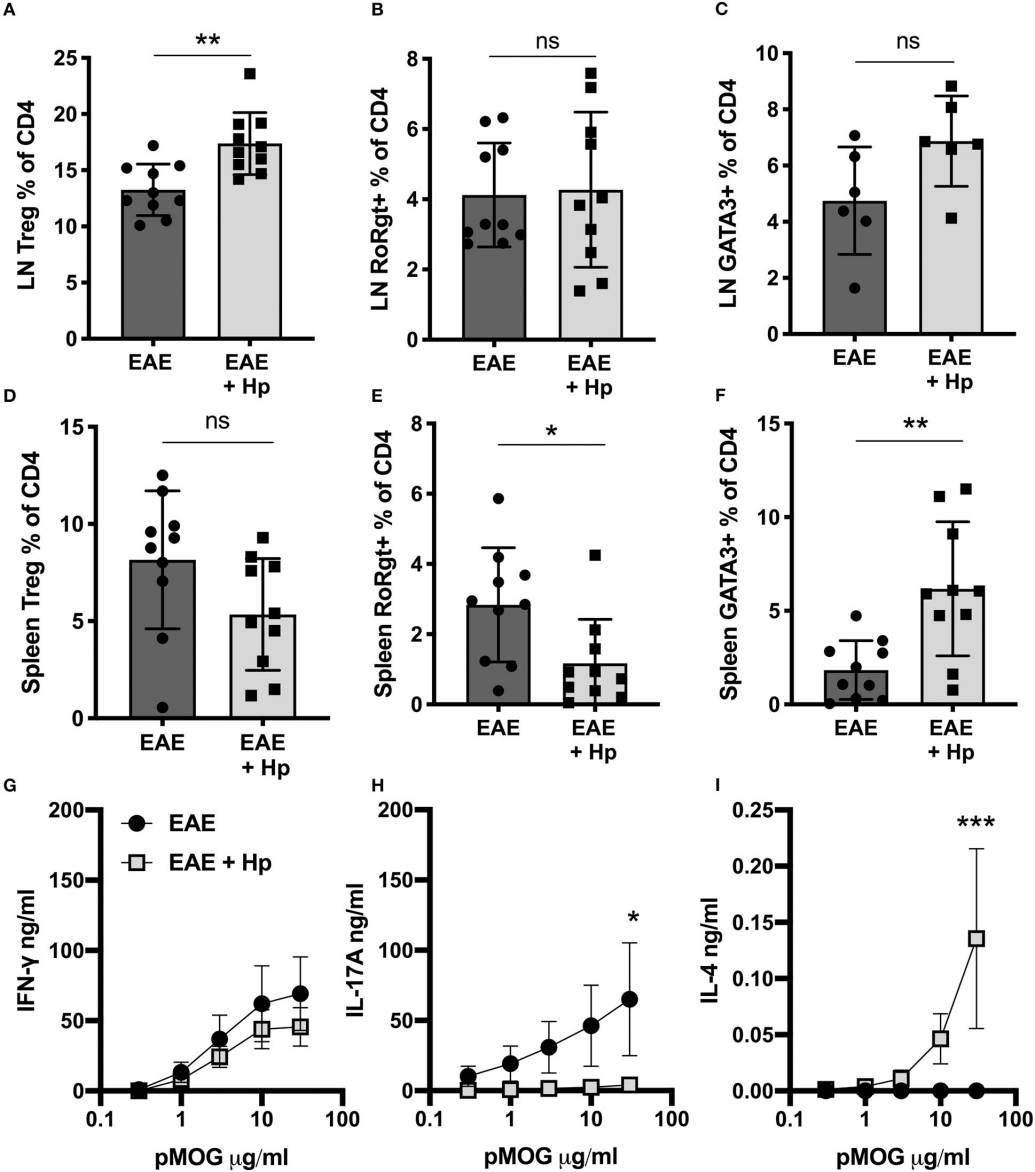
Modes of immune response shift following *H. polygyrus* infection. Female C57BL/6 mice were immunized for EAE on day 0 and were either left untreated (EAE), or received 200 L3 *H. polygyrus* larvae also on day 0 (EAE + Hp). **(A)** Foxp3^+^ Tregs in draining inguinal lymph nodes (LNs), measured by flow cytometry at euthanisation on day 19. **(B)** Percentage of CD4^+^ T cells expressing the Th17 cell marker, RORγt, in inguinal lymph nodes. **(C)** Percentage of CD4^+^ T cells expressing the Th2 marker, GATA3, in inguinal lymph nodes. **(D)** Percentage of Foxp3^+^ Tregs in the spleen. **(E)** Percentage of RORγt^+^ Th17 cells in the spleen. **(F)** Percentage of GATA3^+^ Th2 cells in the spleen. **(G)** MOG-specific responses of spleen cells, IFNγ measured by ELISA in supernatants 72 h post culture. **(H)** MOG-specific responses of spleen cells, IL-17A. **(I)** MOG-specific responses of spleen cells, IFNγ. All data are pooled from two independent experiments, with a total *n* = 10, except for **(C)** in which *n* = 6, and shown are the means ± SEM; **(A–F)** were statistically analyzed by unpaired *t*-tests with Welch's correction is SDs were not equal; **(G–I)** were tested by 2-way ANOVA with Bonferroni's mulitple comparisons tests. *p < 0.05, **p < 0.01, ***p < 0.001.

Infection with *H. polygyrus* was also found to alter the T cell immune profile in the periphery, resulting in localized alterations in major subpopulations. In the (hind limb-draining) inguinal lymph nodes, there was a significant increase in T regulatory cell (Treg) frequency (Figure 2A), and while no change was seen in cells expressing the disease-driving Th17 cell marker RORγt (Figure 2B), cells expressing the Th2 marker GATA3 trended toward an increase (Figure 2C). In the spleen, despite no increase in Tregs (Figure 2D), there was a marked effector T cell skewing with reduced RORγt Th17 (Figure 2E) and expanded GATA3^+^ Th2 (Figure 2F, Supplementary Figure 3).

The shift in effector response mode was even more marked in autoantigen-specific assays, as upon restimulation with the immunizing peptide pMOG_35−55_, the splenocytes from *H. polygyrus-*infected mice secreted lower levels of MOG-specific IL-17A and higher levels of IL-4, whilst MOG-specific IFN-γ was similar between infected and control EAE mice (Figures 2G–I). Overall, these results suggest that infection with *H. polygyrus* induces immunological changes that can mitigate EAE disease, however whether the parasite infection is driving these changes or if the proteins secreted by the adult worm are responsible had yet to be determined.

### *H. polygyrus* Excretory/Secretory Product Marginally Delays EAE Onset

**Figure 3:**
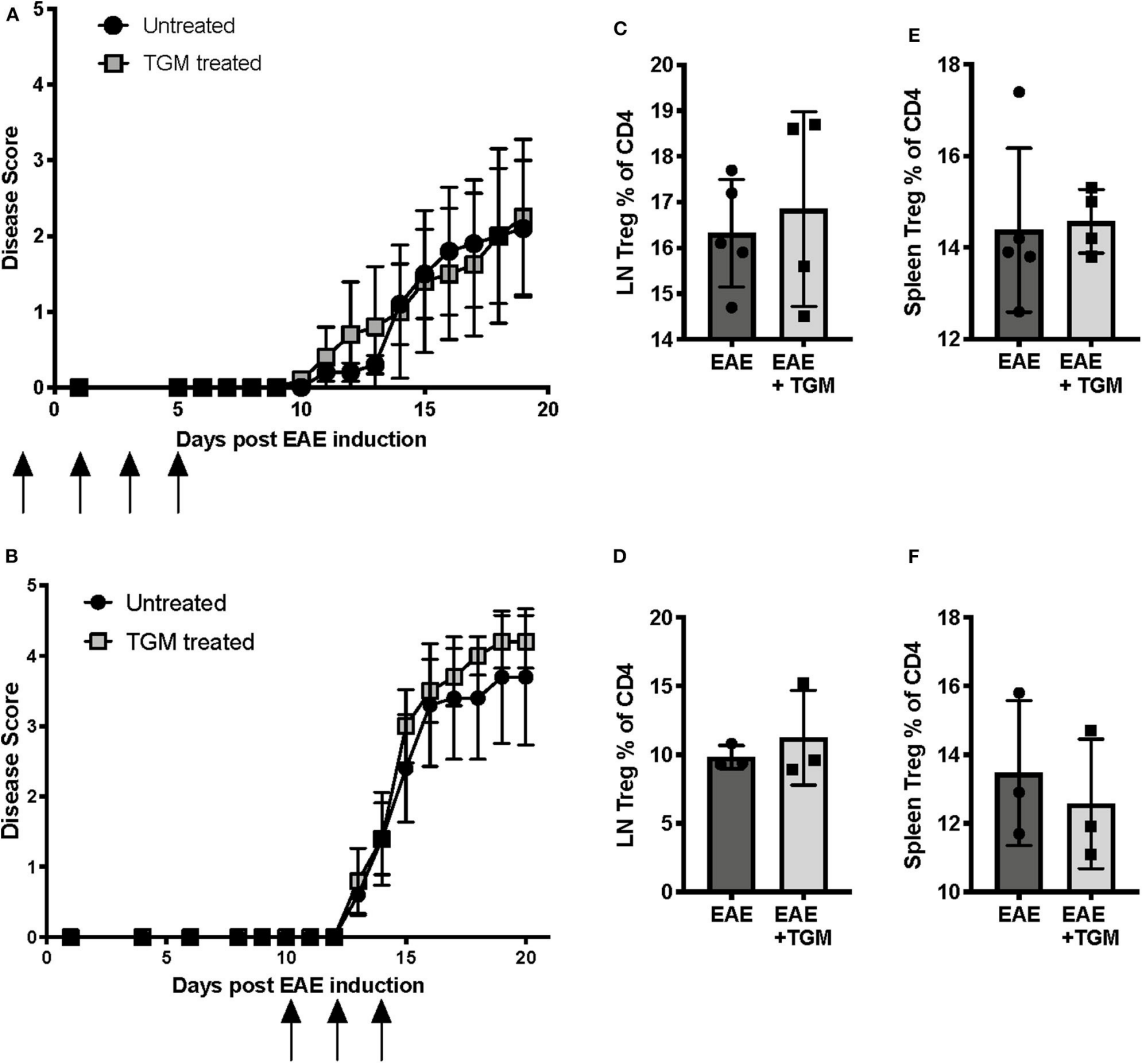
*H. polygyrus* Transforming Growth Factor-β Mimic (*H*p-TGM) does not protect mice from development of EAE. Female C57BL/6 mice were immunized for EAE on day 0 and were either left untreated (EAE) or received *Hp-*TGM (EAE + Hp), spleens and inguinal lymph nodes were assessed for Treg populations at euthanisation at days 19 **(A,C,E)** or 21 **(B,D,F)**. **(A)** *Hp-*TGM was given on days −1, +1, 3, and 5 relative to EAE induction, as indicated by the black arrows. **(B)** *Hp-*TGM was given on days +10, 12, and 14 relative to EAE induction, as indicated by the black arrows. **(C)** Foxp3^+^ Tregs as percentage of all inguinal lymph node CD4^+^ T cells from mice in **(A)**, as measured by flow cytometry at euthanisation. **(D)** Foxp3^+^ Tregs as percentage of all inguinal lymph node CD4^+^ T cells from mice in **(B)**. **(E)** Foxp3^+^ Tregs as percentage of all splenic CD4^+^ T cells from mice in **(A)**. **(F)** Foxp3^+^ Tregs as percentage of all splenic CD4^+^ T cells from mice in **(B)**. Data in **(A**, **B)** each represent individual experiments with no statistical differences between groups, *n* = 5 at the start of both experiments however 2 mice in each group from experiment graphs in **(B)** were culled due to experimental endpoints being reached.

Like many helminth parasites, *H. polygyrus* is known to secrete a number of proteins with immunosuppressive properties (30), and hence we next investigated whether proteins secreted by *H. polygyrus* adult worms are able to suppress EAE disease. As mice infected with *H. polygyrus* showed a significant increase in the frequency of Tregs in the inguinal lymph nodes (Figure 2A), we first hypothesized that one mechanism by which the parasite is offering protection is through the induction of Tregs. Recent studies in our lab have identified a protein, *Hp*-TGM, secreted by *H. polygyrus* that is able to induce *de novo* Tregs by mimicking the action of TGF-β (22). We therefore next tested if administration of this protein can induce Tregs and suppress EAE symptoms. However, when *Hp*-TGM was administered either during disease induction (Figure 3A) or disease onset (Figure 3B), no protection was seen compared to untreated EAE mice. However, we also did not see an expansion of Tregs in either the inguinal lymph nodes (Figures 3C,D) or spleen (Figures 3E,F), suggesting that if Treg induction is the mechanism by which *H. poygyrus* suppresses disease, adminstration of *Hp*-TGM in this way is not effective at inducing Tregs in this model.

**Figure 4:**
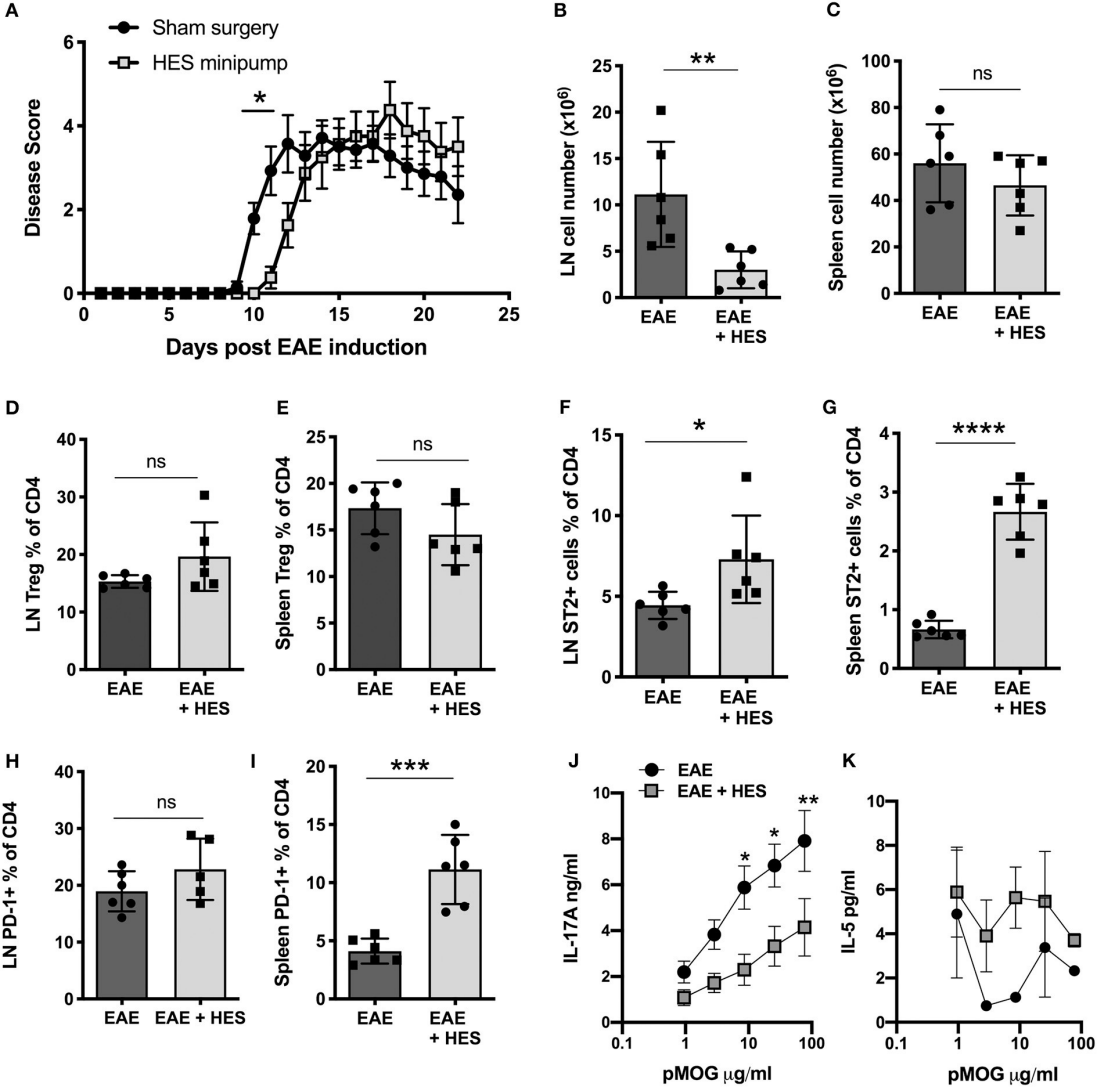
*H. polygyrus* ES administration delays but does not prevent EAE. Female C57BL/6 mice were immunized for EAE on day 0 and received either a sham surgery (EAE) or Alzet mini-osmotic pump surgically implanted i.p, releasing HES at a continuous rate of 0.25 μl per h for 14 days (EAE + HES). (**A)** HES was given by intraperitoneal osmotic minipump 2 days prior to EAE induction. **(B,C)** Total cell numbers in inguinal lymph node **(B)** and spleen **(C)** at day 22. **(D,E)** Foxp3^+^ cell frequencies in inguinal lymph node **(D)** and spleen **(E)**. **(F, G)**. ST2^+^ T cell frequencies in inguinal lymph node **(F)** and spleen **(G)**. **(H,I)** PD-1^+^ T cell frequencies in inguinal lymph node **(F)** and spleen **(G)**. **(J)** MOG-specific responses of spleen cells, IL-17A measured by ELISA at day 22. **(K)** MOG-specific responses of spleen cells, IL-5. Data are from one of two similar independent experiments, with *n* = 6 per group per experiment; **(A,J,K)** were tested by 2-way ANOVA with Bonferroni's mulitple comparisons tests. **(B–I)** were statistically analyzed by unpaired *t*-tests with Welch's correction if SDs were not equal. Shown are the means ± SEM and *p < 0.05, **p < 0.01, ***p < 0.001, and ****p < 0.0001.

Further, we aimed to assess whether the whole *H. polygyrus* excretory/secretory product, HES, can suppress EAE symptoms. To do this, HES was collected from adult *H. polygyrus* as previously described (29) and continuously administered into the peritoneal cavity by osmotic minipumps that were surgically implanted 2 days prior to immunization with pMOG (for initiation of EAE). While HES minipumps only resulted in a slight delay of EAE disease symptoms compared to control sham surgery mice (Figure 4A), there was a significant reduction in the number of cells in the inguinal lymph nodes and a trend to reduction in the spleen (Figures 4B,C), which indicates that HES reduced the local inflammation but could only delay progression of the disease. No significant changes were seen in Treg frequencies in either the lymph node (Figure 4D) or spleen (Figure 4E). Treatment with HES significantly increased the proportion of ST2 expressing CD4^+^ T cells in both the lymph node and spleen, indicating an induction in Th2-type cells (Figures 4F,G). There was also evidence for higher expression of PD-1, in the spleen alone (Figures 4H,I) suggesting that HES-treated mice had a immunological shift toward a more suppressive Th2-type response which is associated with a reduced EAE disease severity.

An immune deviation effect was further supported by the responses of splenocytes restimulated *in vitro* with pMOG, in which HES-treated mice produced lower levels of the disease-driving cytokine IL-17A (Figure 4J), with some suggestion of elevated IL-5 (Figure 4K), a surrogate Th2 cytokine in this experiment. Altogether these results indicate that treating mice with HES during the early EAE induction phase leads to no more than a marginal delay in disease symptoms, perhaps through a modest reduction of cell infiltration to the lymph nodes that drain the site of immunization and dampening of inflammatory IL-17A cytokine responses.

Together the results from *Hp*-TGM and HES administration during EAE indicate that proteins secreted by the parasite may only play a small role in EAE disease suppression by *H. polygyrus*, and that the immune response to the parasite itself may be more crucial.

### Early but Not Chronic Infection With *H. polygyrus* Is Able to Suppress EAE

Because *H. polygyrus* infection on the day of EAE induction protects against disease, while adult secreted immunomodulatory products do not, we postulated that a key factor may be the early events following parasite entry into the intestinal tract. Here, larvae invade the duodenal submucosa for ~7 days, where they molt twice and grow to the adult stage that regains the lumen by day 8, having stimulated a potent type 2 response in the tissues. We therefore compared mice infected, as previously, at the time of EAE induction, with animals that had been infected 28 days earlier, a timepoint known to be immunoregulatory in other models such as airway allergy (31).

**Figure 5:**
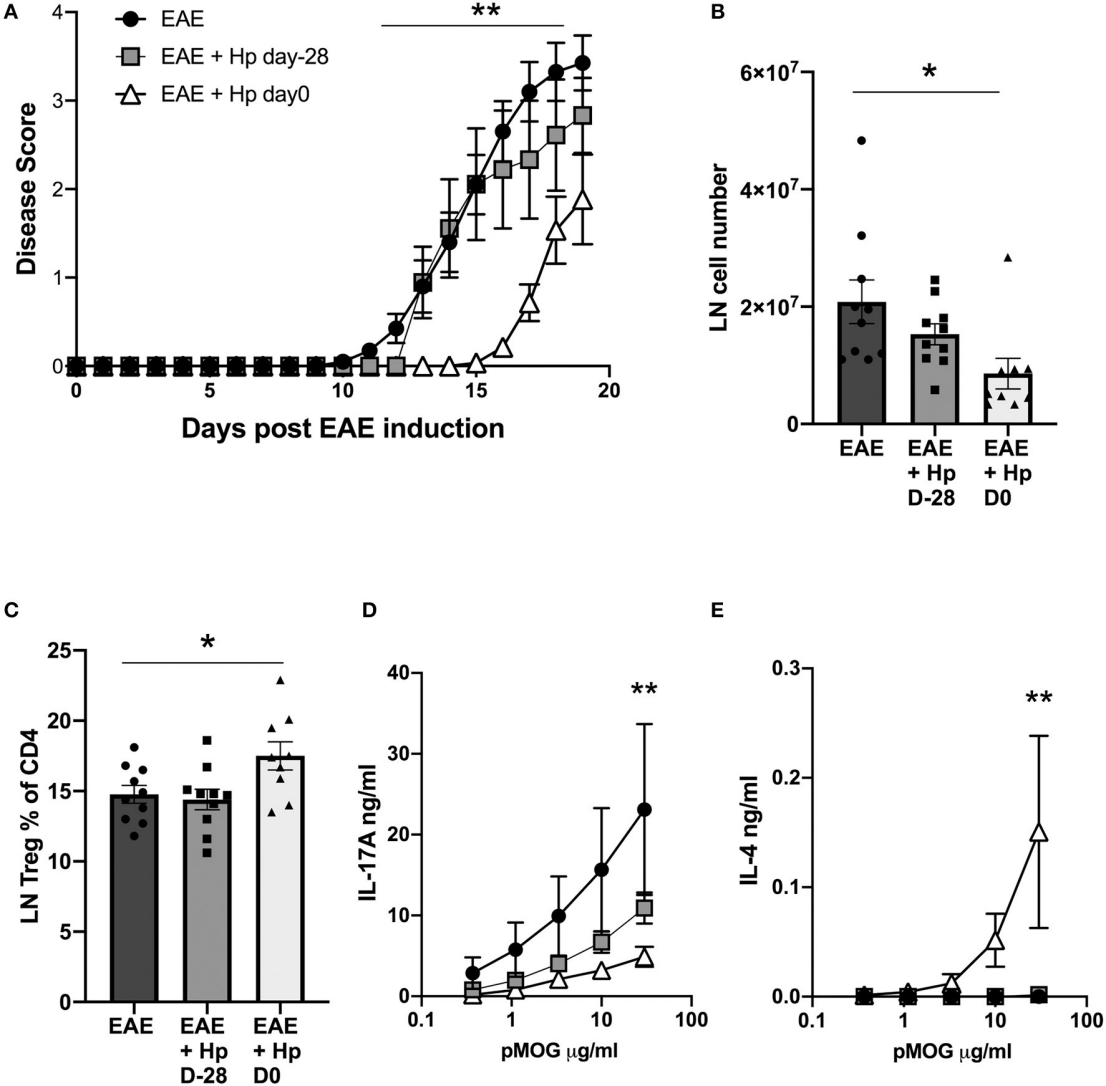
Early, but not chronic, *H. polygyrus* infection is required to inhibit EAE. *H. polygyrus* infection was administered 28 days prior to, or on the day of EAE induction. Female C57BL/6 mice were immunized for EAE on day 0 and were either left untreated (EAE), or received 200 L3 *H. polygyrus* larvae also on day 0 (EAE + Hp D0), or on day−28 (EAE + Hp D-28) prior to EAE immunization. **(A)** Disease course in mice with EAE with or without *H. polygyrus* infection at d0 or day−28. **(B)** Total cell numbers in inguinal lymph nodes at day 19. **(C)** Foxp3^+^ cell frequencies in inguinal lymph node. **(D)** MOG-specific responses of spleen cells, IL-17A measured by ELISA on day 19. **(E)** MOG-specific responses of spleen cells, IL-4. All data are pooled from two independent experiments, with a total *n* = 10, and shown are the means ± SEM; **(A)** was tested by mixed-effects analysis with a multiple comparisons post-tests. **(B,C)** were tested by 1-way ANOVA with Tukey's multiple comparisons tests. **(D,E)** were tested by 2-way ANOVA with Bonferroni's multiple comparisons tests. Shown are the means ± SEM *p < 0.05, **p < 0.01.

When mice infected at the different time points were compared for the course of disease, it was clear that animals bearing a chronic infection showed little protection against disease, unlike mice that were infected on the day of EAE induction and experiencing the early, tissue-invasive phase of infection (Figure 5A). As previously, co-incident infection with EAE induction reduced cell infiltration into the inguinal lymph nodes (Figure 5B), and increased Treg frequencies (Figure 5C), neither of which were observed in chronically-infected mice. More notably, the pMOG-specific cytokine responses of spleens from mice infected 28 days prior to EAE induction did not differ significantly from the uninfected group, while simultaneously-infected mice showed a significant reduction in pMOG-specific IL-17A and sharply elevated pMOG-specific IL-4 responses (Figures 5D,E). These results indicate that *H. polygyrus* is most able to suppress EAE disease during the tissue-invasive stages of infection and during this phase MOG-specific T cells are polarized toward the Th2 mode which has been found to offer protection in previous studies of the EAE model (19, 20).

### *H. polygyrus* Disease Suppression Is IL-4Rα-Dependent

**Figure 6:**
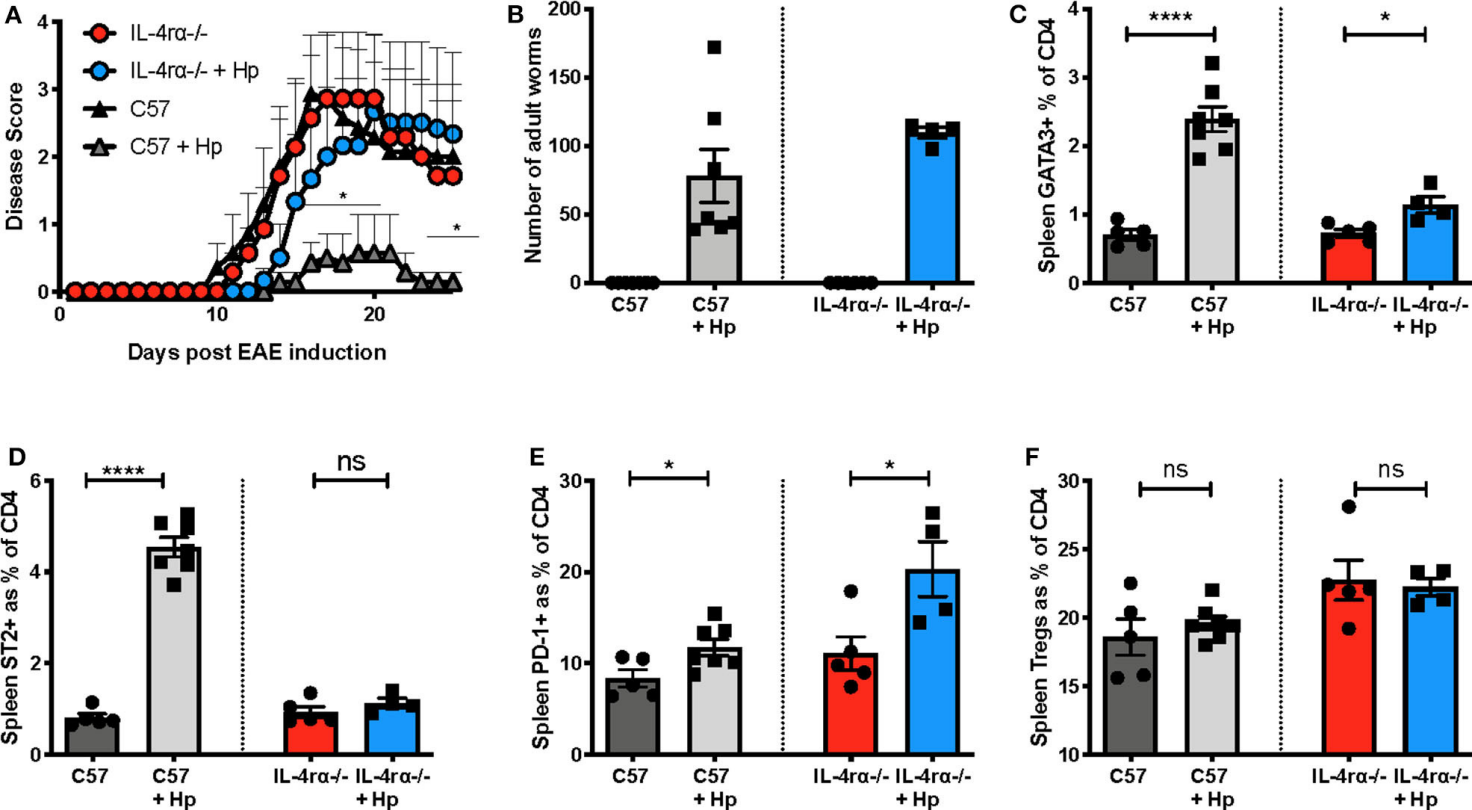
*H. polygyrus* fails to protect against EAE in IL-4Rα-deficient mice. Female C57BL/6 or IL-4Rα-deficient mice were immunized for EAE on day 0 and were either left untreated or received 200 L3 *H. polygyrus* larvae also on day 0 (+Hp). **(A)** Disease course of EAE in uninfected and infected C57BL/6 wild-type and IL-4Rα-deficient mice. **(B)** Adult worm burdens in infected C57BL/6 wild-type and IL-4Rα-deficient mice at the time of euthanisation, day 26. **(C)** Frequency of CD4^+^ T cells expressing the Th2 marker, GATA3, in spleen at day 25 as measured using flow cytometry. **(D)** Frequency of CD4^+^ T cells expressing ST2 in spleen. **(E)** Frequency of CD4^+^ T cells expressing PD1 in spleen. **(F)** Frequency of Foxp3^+^ Tregs in the spleen. Data are from one of two similar independent experiments with *n* = 4–6 per group; **(A)** was tested by a 2-way ANOVA with Bonferroni's multiple comparisons test. **(C–F)** were statistically analyzed by unpaired *t*-tests with Welch's correction if SDs were not equal. Shown are the means ± SEM and *p < 0.05 and ****p < 0.0001.

In view of the marked skew toward Th2 during EAE in mice infected with *H. polygyrus*, including induction of pMOG-specific IL-4 responses, we aimed to assess whether signalling through the IL-4Rα plays a critical role in EAE disease suppression by this helminth. We therefore tested mice that were deficient for IL-4Rα (IL-4Rα^−/−^), the shared receptor component that transduces signals for both IL-4 and IL-13. In these gene-targeted mice, *H. polygyrus* was unable to significantly suppress EAE disease unlike C57BL/6 wild-type control mice (Figure 6A) even though their worm load was slightly higher (Figure 6B) and this lack of protection was accompanied by minimal Th2 and ST2^+^ CD4 T cell expansion compared to wild-type mice (Figures 6C,D). However, both IL-4Rα^−/−^ and C57BL/6 mice showed an increase in PD-1^+^ CD4 T cells with *H. polygyrus* infection, suggesting that despite their association with immune down-regulation, this cell subset is not involved in dampening EAE in this model (Figure 6E). Neither genotype showed any rise in Treg frequency at this time-point (day 26 post-infection), and while the baseline Treg proportions were actually higher in IL-4Rα^−/−^ mice (Figure 6F), there was no correlation with protection, as disease severity was similar between IL-4Rα^−/−^ and wild-type mice. Together, these results indicate that the ability to mount a strong Th2 response to *H. polygyrus* is central to the suppression of EAE disease by the parasite, and the timing of the anthelminthic Th2 response is pivotal for suppression of disease.

## Discussion

Helminth parasites have been hypothesized as important environmental regulators for immune tolerance in both model systems and in human populations (2, 17, 32), resulting in numerous studies assessing the use of helminths or their products as therapeutic agents in the fight against autoimmune disease. In the current study we used a natural mouse parasite *H. polygyrus*, a model for human intestinal helminth infection, to understand the mechanism(s) by which helminths are able to suppress an animal model of multiple sclerosis with the aim to further understand the immune pathways involved in suppressing autoimmune disease.

Infection with *H. polygyrus* is known to induce many immunoregulatory pathways including Tregs, alternatively activated macrophages and regulatory dendritic cells, as well as regulatory B cells acting through IL-10 (33), suggesting various potential mechanisms by which *H. polygyrus* is capable of ameliorating inflammatory diseases (34). Here we show that infection with *H. polygyrus* protects mice from developing severe EAE and that this disease suppression is in fact mediated through IL-4R signaling and Th2 immune deviation rather than a Treg mechanism, as IL-4Rα^−/−^ mice infected with *H. polygyrus* developed EAE symptoms similar to uninfected mice. We also show that some of this protection is not limited to infection with the live parasite, as administration of the excretory/secretory product, HES, is able to delay disease onset. Although these mice do succumb to EAE, they also show reduced cell infiltration in the immunization draining lymph nodes and reduced pMOG-specific IL-17A release.

Interestingly, chronic *H. polygyrus* infection (day 28) prior to EAE induction was not protective, although may limit the disease severity which indicates active immune deviation to Th2 may be essential to suppress developing Th17/Th1 responses in the EAE model. Furthermore, day 28 of infection coincides with adult *H. polygyrus* residing in the gut, where they secrete HES proteins, therefore these results are consistent with HES administration only having a limited effect on the EAE disease severity. In contrast, our finding that protection coincides with the early stages of active infection are consonant with recent reports from another laboratory, that infection of mice at the peak of EAE disease results in a rapid amelioration of symptoms, accompanied by evidence of downregulation within the myeloid compartment (24, 25). During the initial 7 days of infection, serum IL-4 reaches a peak and declines thereafter (23), supporting our findings that the early phase of infection is the most protective. Further, type 2 innate lymphoid cell (ILC2) responses are at their most prominent in early infection (35, 36), and IL-4/IL-13 from this subset is likely to contribute to the Th2 skewing observed in our model.

Our results are also consistent with other helminth infections that have been shown to potently modulate EAE such as *S. mansoni* (18), *T. peusdospiralis* (37), *F. hepatica* (21), *Trichinella spiralis* (38), and *Taenia crassiceps* (39) with protection in these infections mostly being attributed to Th2 immune deviation and suppressed levels of pMOG-specific TNF, IFN-γ, and IL-17A. These studies also highlighted other cell populations as potential suppressors of T cell activation such as alternatively activated macrophages (AAM) and myeloid-derived suppressor cells (MDSC) that regulate CNS infiltration and EAE progression. During *H. polygyrus* infection AAM are induced by Th2 cells after epithelial damage alerts the immune system of parasite infection, resulting in IL-4 release (40) and phenotypic shifts in both peritoneal and CNS macrophage populations (25). It is therefore possible that myeloid cells are also crucial for EAE protection with *H. polygyrus* as it is known that AAM and MDSCs are able to ameliorate EAE (41, 42) and therefore this may be one of the ways in which IL-4 signaling is protective in *H. polygyrus-*infected EAE mice.

In some instances the suppressive effect of helminth infection on EAE can be mimicked by the parasites' excretory/secretory products (ES). However, in the current study we found HES was only able to delay disease onset indicating that the immunological changes produced by HES were not strong enough to recapitulate the effect seen with live parasite infection. In contrast, a study with *Taenia crassiceps* ES (TcES), which showed that in addition to Th2-deviation, ES was able to sequester inflammatory cells into the periphery and therefore inhibit the induction of EAE by preventing trafficking of cells to the CNS (43), a mechanism that could well underpin the protective ability of live *H. polygyrus*. Soluble egg antigens (SEA) from *S. japonicum* and *T. spiralis* ES also ameliorate EAE disease in a way that was similar to whole parasite infection, protection that was related to a Th2 shift in both the periphery and CNS (19, 44).

Although HES treatment in our study did not offer long-term protection from EAE, a significant reduction in pMOG-specific IL-17A indicated that some aspects of autoimmunity are suppressed, although overall disease symptoms eventually appear regardless. Consistent with HES, a moderate reduction in IL-17A was seen in mice with a chronic *H. polygyrus* infection where adult *H. polygyrus* parasites would be present in the intestine secreting the HES products, indicating these ES proteins alter the autoimmunity status of cells in the periphery but ultimately disease symptoms progress. Interestingly, IL-17A is thought to downregulate the tight-junction proteins therefore increasing the permeability of the BBB and allowing CNS infiltration to occur (45). In addition, IL-17A is also thought to recruit activated neutrophils and monocytes into the CNS, facilitating further damage and demyelination which exacerbates symptoms of EAE disease further (46). Given that HES and chronically infected *H. polygyrus* mice had a reduced IL-17A response and a delay or slight reduction in EAE symptoms in our study, it is possible that this reduction suppressed early EAE disease and was then overcome as disease progressed further. It is also possible that the lack of efficacy in our study may be due to the dose of HES used, we chose 2 μg/mouse/day by continuous infusion while other studies administered up to 250 μg of protein by subcutaneous or intraperitoneal injection every other day (43, 47, 48).

While many studies have identified Th2-immune deviation as the dominant mechanism by which helminths suppress EAE disease, there is also mounting evidence that IL-33 signaling also plays a central role. One study looked at the role of the IL-33 receptor subunit, ST2, which when knocked out abrogates resistance to EAE in the partially resistant BALB/c mouse strain. The adoptive transfer of pMOG-specific CD4^+^ T cells from ST2^+/+^ mice were unable to induce EAE in ST2^−/−^ mice indicating signaling through the IL-33 receptor on CD4^+^ T cells is suppressive in the EAE model (49). In the case of *F. hepatica* ES (FHES), administration induced Th2 responses, but disease protection was independent of IL-4, IL-10 and Tregs, although it was IL-33-dependent and the transfer of FHES-induced eosinophils conferred protection in the EAE model (50). Together these studies suggest that in some helminth infection models signaling through IL-33 may be essential for EAE disease protection. In the current study we identified that both HES and infection with *H. polygyrus* induced ST2 expression on CD4^+^ T cells and interestingly in the IL-4Rα^−/−^ mice, which were not protected with *H. polygyrus*, there was very little expression of ST2 suggesting that IL-33 signaling may also play a role in EAE protection in this model.

The programmed cell-death (PD)-1/PD-L1 pathway is extensively studied in cancer research; however this tolerogenic pathway is also critical for maintaining homeostasis and deficits can lead to the development of autoimmunity. Binding of PD-1 on the surface of T cells with its ligands, PD-L1 or PD-L2 which are widely expressed on both haematopoetic and non-haematopoetic cell types, suppresses T cell activation and in the presence of TGF-β may promote *de novo* generation of Tregs (51). Therefore, expression of PD-1 and PD-L1/PD-L2 during helminth infection may result in suppressed T cell responses which is not only beneficial for the host but may also suppress bystander inflammatory diseases such as EAE. In the current study, CD4^+^ T cells from *H. polygyrus* infected and HES-treated mice expressed higher levels of PD-1 compared to control EAE mice, indicating that expression of this marker may also contribute to the disease suppression seen. It is important to note however that PD-1 expression was also elevated in *H. polygyrus-*infected IL-4Rα^−/−^ mice, but these animals were not significantly protected from EAE, indicating that PD-1 expression may only have a small role in the mechanism of *H. polygyrus*.

The CNS is now recognized to have a close relationship with the gastrointestinal tract, and therefore helminth infections within the gut may disrupt the normal gut-brain axis and interfere with disease progression in a more indirect manner. Studies have shown that alteration of the gut microbiota can have profound effects on EAE disease development, with antibiotic-mediated depletion of intestinal microbes impairing the development of EAE through an IL-10-dependent mechanism (52). These results indicate that changes in the gut can have striking effects on CNS disease development and also be mediated through an alteration in the peripheral immune response. There are also significant changes to the gut microbiota with *H. polygyrus* infection (53–55), which are unlikely to recur with the systemic administration of ES products, further contributing to the requirement for live infection for amelioration of disease. In our study with *H. polygyrus* this could also be one of the key differences in the level of disease protection seen with HES compared to infection.

In conclusion our study has further supported work by other researchers assessing the effect of intestinal helminths in the amelioration of an animal model of MS. Notably, we identified that infection with *H. polygrus* was able to suppress EAE in an IL-Rα-dependent manner, with further evidence to suggest that a lack of IL-4 signaling also ablates any disease protection mediated through the IL-33/ST2 receptor. Perhaps surprisingly, it was found that ES products from *H. polygyrus* were unable to mimic disease protection seen with live infection unlike results from other helminths. Altogether our results indicate that both a strong Th2 shift as well as parasite mediated damage in the small intestine is required to ameliorate EAE with *H. polygyrus* infection.

## Data Availability Statement

The raw data supporting the conclusions of this article will be made available by the authors, without undue reservation.

## Ethics Statement

The animal study was reviewed and approved by University of Glasgow Animal Welfare and Ethical Review Board.

## Author Contributions

MW, CJ, JG, JK, and RO'C performed the experiments. SA and RM supervised the laboratory work. MW drafted the manuscript which was edited by CJ and RM. All authors contributed to the article and approved the submitted version.

## Conflict of Interest

The authors declare that the research was conducted in the absence of any commercial or financial relationships that could be construed as a potential conflict of interest.

## Supplementary Material

Supplementary Figure 1 *H. polygyrus* suppresses cellular infiltration in mouse model of EAE disease. Female C57BL/6 mice were immunised for EAE on day 0 and were either left untreated (EAE), or received 200 L3 *H. polygyrus* larvae also on day 0 (EAE + Hp). **(A)** Spinal cord CD4^+^ T cell infiltration at euthanisation on day 19, relative to the number of glial cells. **(B)** Spinal cord macrophage infiltration (CD11b^+^CD45^hi^ cells as gated detailed in Supplementary Figure 2) at euthanisation on day 19, relative to the number of glial cells. Data are pooled from two independent experiments, with a total *n* = 10, and show arithmetic means and standard errors. Data were analyzed by Mann-Whitney nonparametric test **(C,D)**, ^*^*p* < 0.05.

Supplementary Figure 2 Gating strategy for spinal cord infiltrating macrophages. Female C57BL/6 mice were immunized for EAE on day 0 and were either left untreated (EAE), or received 200 L3 *H. polygyrus* larvae also on day 0 (EAE + Hp). Within the gate of sinle live leukocytes, CD45^+^ spinal cord cells were gated into non-myeloid cells (CD45^+^CD11b^−^), microglia (CD45^int^CD11b^+^) and infiltrating macrophages (CD45^+^CD11b^+^). Further gating from non-myeloid cells into T cells (CD3^+^) and finally CD4 (CD4^+^) and CD8 (CD8^+^) T cells.

Supplementary Figure 3 Alterations in cell numbers following *H. polygyrus* infection in EAE mice. Female C57BL/6 mice were immunised for EAE on day 0 and were either left untreated (EAE), or received 200 L3 H. polygyrus larvae also on day 0 (EAE + Hp). **(A)** Cell numbers in draining inguinal lymph nodes (LNs), measured by flow cytometry at euthanisation on day 19. **(B)** Cell numbers in spleen, measured by flow cytometry at euthanisation on day 19. All data are in **(A)** are from a single experiment (*n* = 5/group) and in **(B)** pooled from two independent experiments, with a total n = 10/group, and shown are the means ± SEM; **(B)** was statistically analysed by unpaired *t*-test with Welch's correction for unequal variances, ^*^*p* < 0.05, ^**^*p* < 0.01.
