# Supplementary figures and images for: The Helminth Parasite Heligmosomoides polygyrus Attenuates EAE in an IL-4Rα-Dependent Manner
Source: Front Immunol. 2020 Sep 29;11:1830. doi: 10.3389/fimmu.2020.01830 (PMC7552805; doi:10.3389/fimmu.2020.01830)

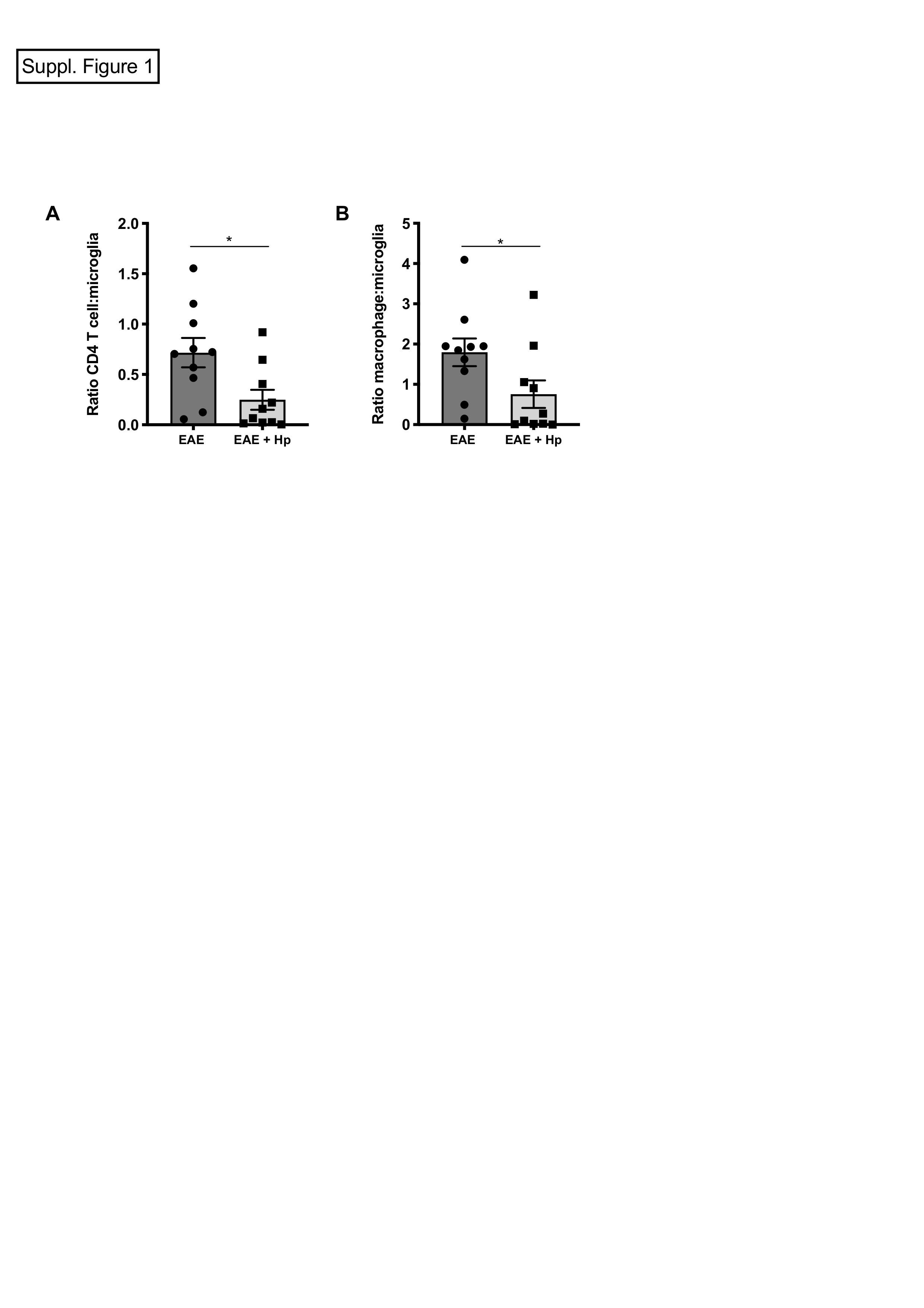

Supplement: Supplementary Figure 1 — H. polygyrus suppresses cellular infiltration in mouse model of EAE disease. Female C57BL/6 mice were immunised for EAE on day 0 and were either left untreated (EAE), or received 200 L3 H. polygyrus larvae also on day 0 (EAE + Hp). (A) Spinal cord CD4+ T cell infiltration at euthanisation on day 19, relative to the number of glial cells. (B) Spinal cord macrophage infiltration (CD11b+CD45hi cells as gated detailed in Supplementary Figure 2) at euthanisation on day 19, relative to the number of glial cells. Data are pooled from two independent experiments, with a total n = 10, and show arithmetic means and standard errors. Data were analyzed by Mann-Whitney nonparametric test (C,D), *p < 0.05. [file Data_Sheet_1.zip › Supplementary Figure S1.png]

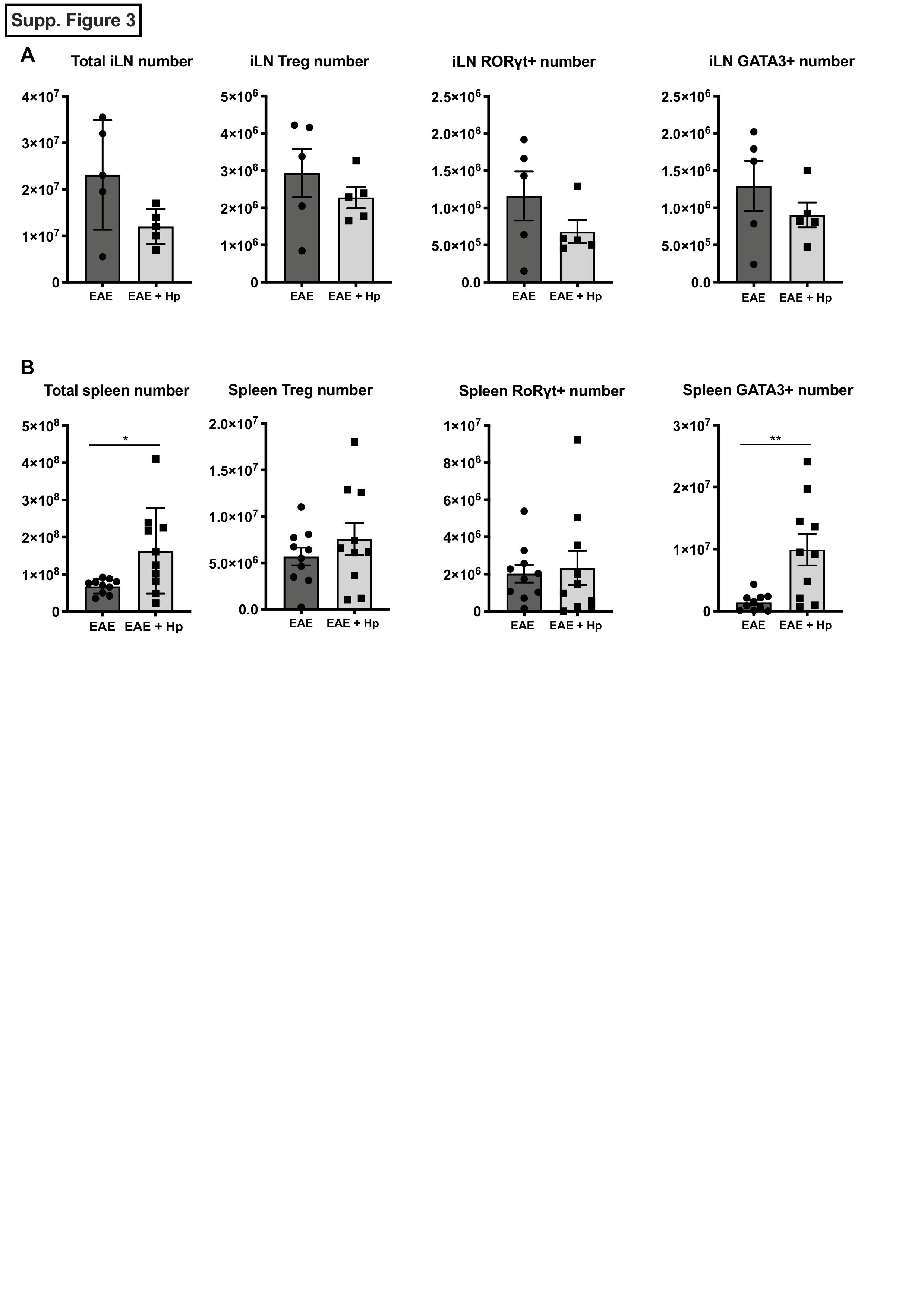

Supplement: Supplementary Figure 1 — H. polygyrus suppresses cellular infiltration in mouse model of EAE disease. Female C57BL/6 mice were immunised for EAE on day 0 and were either left untreated (EAE), or received 200 L3 H. polygyrus larvae also on day 0 (EAE + Hp). (A) Spinal cord CD4+ T cell infiltration at euthanisation on day 19, relative to the number of glial cells. (B) Spinal cord macrophage infiltration (CD11b+CD45hi cells as gated detailed in Supplementary Figure 2) at euthanisation on day 19, relative to the number of glial cells. Data are pooled from two independent experiments, with a total n = 10, and show arithmetic means and standard errors. Data were analyzed by Mann-Whitney nonparametric test (C,D), *p < 0.05. [file Data_Sheet_1.zip › Supplementary Figure S3.png]
